# High Levels of TNF-α and TIM-3 as a Biomarker of Immune Reconstitution Inflammatory Syndrome in People with HIV Infection

**DOI:** 10.3390/life11060527

**Published:** 2021-06-05

**Authors:** Lucero A. Ramon-Luing, Ranferi Ocaña-Guzman, Norma A. Téllez-Navarrete, Mario Preciado-García, Dámaris P. Romero-Rodríguez, Enrique Espinosa, Gustavo Reyes-Terán, Leslie Chavez-Galan

**Affiliations:** 1 Laboratory of Integrative Immunology, Instituto Nacional de Enfermedades Respiratorias “Ismael Cosío Villegas”, Mexico City 14080, Mexico; ramonluing@yahoo.com.mx (L.A.R.-L.); ranferi.og@gmail.com (R.O.-G.); norma.tellez@gmail.com (N.A.T.-N.); mario77.preciado@gmail.com (M.P.-G.); hector.enrique.espinosa@gmail.com (E.E.); 2 Flow Cytometry Core Facility, Instituto Nacional de Enfermedades Respiratorias “Ismael Cosío Villegas”, Mexico City 14080, Mexico; damaquim@gmail.com; 3 Center for Infectious Diseases Research (CIENI), Instituto Nacional de Enfermedades Respiratorias “Ismael Cosío Villegas”, Mexico City 14080, Mexico; gustavo.reyesteran@gmail.com

**Keywords:** HIV, IRIS, TNF-α, ADAM, TIM-3

## Abstract

Immune reconstitution inflammatory syndrome (IRIS) is an exacerbated immune response that can occur to HIV+ patients after initiating antiretroviral therapy (ART). IRIS pathogenesis is unclear, but dysfunctional and exhausted cells have been reported in IRIS patients, and the TIM-3/Gal-9 axis has been associated with chronic phases of viral infection. This study aimed to evaluate the soluble levels of TIM-3 and Gal-9 and their relationship with IRIS development. TIM-3, Gal-9, TNF-α, IFN-γ, IL-6, TNFR1, TNFR2, E-cadherin, ADAM10, and ADAM17 were measured to search for IRIS-associated biomarkers in plasma samples from 0-, 4-, 8-, 12-, and 24-weeks after ART initiation of 61 HIV+ patients (15 patients developed IRIS, and 46 did not). We found that patients who developed IRIS had higher levels of TIM-3 [median 4806, IQR: 3206–6182] at the time of the IRIS events, compared to any other follow-up time evaluated in these patients or compared with a control group of patients who did not develop IRIS. Similarly, IRIS patients had a higher TNF-α level [median 10.89, IQR: 8.36–12.34] at IRIS events than any other follow-up time evaluated. Other molecules related to the TIM-3 and TNF-α pathway (Gal-9, IL-6, IFN-γ, TNFR1, TNFR2, ADAM-10, and ADAM-17) did not change during the IRIS events. In conclusion, our data suggest that a high level of soluble TIM-3 and TNF-α could be used as an IRIS biomarker.

## 1. Introduction

Immune reconstitution inflammatory syndrome (IRIS) is a clinical complication of some patients infected with the human immunodeficiency virus (HIV) after starting antiretroviral therapy (ART). IRIS is characterised by the release of proinflammatory cytokines and tissue inflammation, and it is related to a well-identified coinfection [1].

Although several aspects of IRIS pathogenesis remain unclear, two clinical parameters have been related to IRIS development: (1) severe CD4+ lymphopenia and (2) opportunistic diseases that lead to a dysregulated immune response (usually characterised by persistent T cell activation, which favours T cell exhaustion) [2,3,4]. IRIS is diagnosed using the criteria from the International Network for the Study of HIV-associated IRIS (INSHI) [5]. Several studies have focused on identifying biomarkers for IRIS prognosis or diagnosis, but mainly on tuberculosis (TB)-IRIS [1]. 

Plasma could be a valuable resource to look for potential biomarkers of inflammation associated with IRIS [4,5]. It is well documented that molecules associated with T cell exhaustion, including T cell immunoglobulin and mucin domain 3 (TIM-3), lymphocyte-activation gene 3 (LAG-3), and cytotoxic T-lymphocyte-associated antigen 4 (CTLA-4), are increased in some viral infections, such as HIV and hepatitis C, both during the acute and chronic phases [6,7]. The increased frequency of TIM-3+ T cells in HIV+ patients is associated with T cell dysfunction, and blocking the TIM-3 pathway in T cells restores their function to produce IFN-γ, tumour necrosis factor alpha (TNF-α), and interleukin 6 (IL-6) [7]. Galectin-9 (Gal-9) is one of the most relevant ligands of TIM-3; in HIV+ patients, a high plasma level of soluble Gal-9 (sGal-9) has been associated with high viral load [8]. 

This study was conducted to identify if soluble levels of TIM-3 (sTIM-3) and sGal-9 are affected in the plasma of HIV+ patients who developed IRIS. Furthermore, we evaluated if proinflammatory cytokines, such as IFN-γ, TNF-α, and IL-6, and receptors and enzymes associated with a proinflammatory profile are affected in HIV+ patients who develop IRIS, in order to identify a specific profile predictive of this condition. Our results suggest that high plasma levels of sTIM-3 and sTNF-α are characteristic of IRIS onset and could be considered as IRIS biomarkers in HIV+ patients.

## 2. Materials and Methods

### 2.1. Patients

Sixty-one male subjects with HIV, naïve to ART, were selected from a previously described cohort [9]. Patients were selected based on the availability of plasma samples at baseline before ART initiation (hereafter called week 0). According to the International Guidelines 2004 [10], all of them initiated ART and showed viral control at/or before week 24 of ART initiation. Plasma samples corresponded to 0-, 4-, 8-, 12, and 24-weeks after ART initiation. Additionally, in HIV+ patients who developed IRIS, one sample was obtained at the time of the IRIS event (referred to as IRIS in graphics). We included 15 patients that developed IRIS (hereafter called HIV+IRIS) and 46 patients that did not develop IRIS (hereafter called HIV+). Data of healthy donors paired by sex and age to the HIV+ and HIV+IRIS patients were included, as shown in each graphic as a red line; some data were published by our group [11], and others are unpublished data taken from our group laboratory (Table S1). 

### 2.2. IRIS Diagnosis

According to the consensus of attending physicians (including at least 3 persons of which 2 consistently participated in all decisions), IRIS diagnosis was made based on previously published criteria, where IRIS was defined as the appearance of signs or symptoms consistent with inflammation, appearance of new opportunistic infections (OI), or worsening of previously controlled OI with satisfactory ART [5,10]. All HIV+IRIS patients included in the present study developed unmasking IRIS. 

### 2.3. Soluble Levels Measurements in Plasma Samples by Enzyme-Linked Immunosorbent Assay (ELISA) 

Plasma samples were stored at −70 °C until analysis. Soluble levels of TIM-3, Gal-9, E-cadherin, TNFR1, TNFR2 (provided by R&D Systems, Minneapolis, USA), TNF-α, IFN-γ, IL-6 (provided by BioLegend, San Diego, CA, USA), ADAM10, and ADAM17 (provided by Cloud-Clone Corp., Houston, TX, USA) were quantified by ELISA following the manufacturer’s protocols. All proteins were quantified by comparison with the corresponding standard curve. 

### 2.4. Statistical Analysis

Data are shown as median values and interquartile range (IQR, 25–75). One-way ANOVA with post hoc Dunnett’s test was used to compare IRIS events with the different follow-up times, and Mann–Whitney U test to compare HIV+ or HIV+ IRIS patients at each follow-up (0-, 4-, 8-, 12-, and 24-weeks post-ART). *p*-values <0.05 were considered statistically significant (GraphPad Software, Inc., San Diego, CA, USA).

## 3. Results

### 3.1. Baseline Characteristics of Study Populations

We included a total of 61 participants, 15 subjects with a diagnosis of unmasking IRIS (HIV+IRIS, 25%) and 46 subjects with no IRIS diagnosis (HIV+, 75%). The median age was 34 years (IQR 29–39). Baseline laboratory variables (HIV plasma viral loads, CD4+ T cell and CD8+ T cell counts, and CD4/CD8 ratio) were analysed across the follow-up times, and there were no differences between groups (Table 1). 

The median time to IRIS diagnosis was 7.5 weeks; only three (20%) patients developed IRIS at 20 weeks after starting ART. Regarding the IRIS-associated pathogen, herpes viruses were the most frequent (47%), including herpes zoster virus (HZV), herpes simplex virus (HSV), and cytomegalovirus, followed by mycobacterial (27%), molluscum contagiosum, and eosinophilic folliculitis (20%) as other dermatological manifestations (Table 2).

### 3.2. sTIM-3 Increased in HIV+IRIS Patients at IRIS Onset

**Figure 1:**
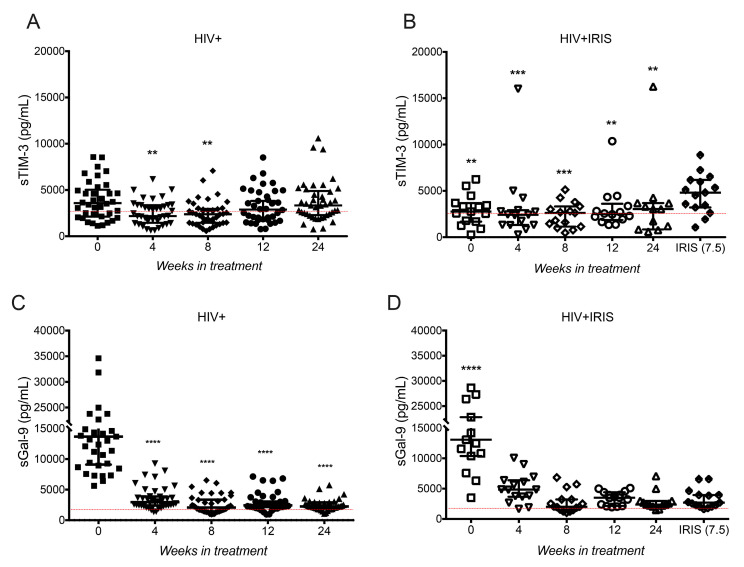
sTIM-3 increases at IRIS events. Plasma levels of sTIM-3 (**A**,**B**) and sGal-9 (**C**,**D**) in HIV patients (**A**,**C**) at 0- (■), 4- (▼), 8- (♦), 12- (●), 24- (▲) weeks after ART, and HIV+IRIS patients (**B**,**D**) at 0- (☐), 4- (▽), 8- (◇), 12- (○), 24- (△) weeks after ART, and IRIS episodes (⬘). The red line represents the mean of healthy donors. Post hoc Dunnett’s test compares the control (0 W) in the HIV+ group or IRIS with the HIV+IRIS group. ** *p* < 0.01, *** *p* < 0.001, **** *p* < 0.0001.

We evaluated plasma levels of sTIM-3 and sGal-9 to assess possible alterations of these markers in HIV+IRIS patients. sTIM-3 significantly decreased in HIV+ patients compared to week 0 [3582, 2120–5028] and at week 4 [2183, 1454–3358, *p* = 0.0048] and 8 [2376, 1451–2952, *p* = 0.0025], but at weeks 12 and 24, sTIM-3 levels were similar (Figure 1A). 

In HIV+IRIS patients, sTIM-3 levels were significantly higher at IRIS onset compared to week 0 [2762, 1683–3686, *p* = 0.0087], 4 [2387, 1309–2819, *p* = 0.0003], 8 [2619, 1138–3319, *p* = 0.0004], 12 [2464, 1784–3091, *p* = 0.0021], and 24 [3031, 833.4–3664, *p* = 0.0012] (Figure 1B). 

Consistent with previous reports [12,13], sGal-9 levels were higher in HIV+ patients at baseline and decreased after ART initiation from week 4 up to week 24 (*p* = 0.0001 in all cases) (Figure 1C). HIV+IRIS patients showed a similar profile to HIV+ patients, with a significant decrease of sGal-9 levels in all follow-up points compared to baseline (*p* = 0.0001 in all cases) (Figure 1D). These data show that only sTIM-3 level, not sGal-9, increases at IRIS events.

### 3.3. IFN-γ Levels Did Not Increase in HIV+IRIS Patients at IRIS Onset

**Figure 2:**
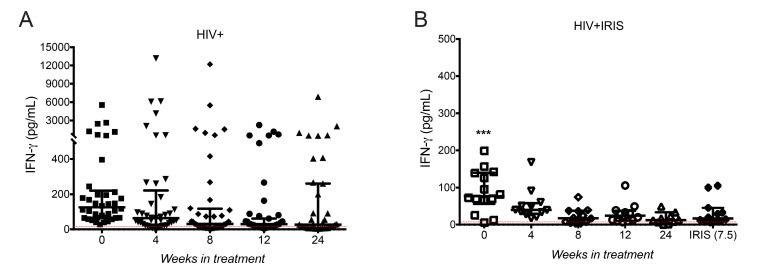
Circulating levels of IFN-γ did not increase at IRIS events. IFN-γ plasma levels in HIV+ (**A**) at 0- (■), 4- (▼), 8- (♦), 12- (●), 24- (▲) weeks after ART, and HIV+IRIS patients (**B**) at 0- (☐), 4- (▽), 8- (◇), 12- (○), 24- (△) weeks after ART, and IRIS episodes (⬘). The red line represents the mean value of healthy donors. Post hoc Dunnett’s test compares the control (0 W) of the HIV+ group or IRIS group with the HIV+IRIS group. *** *p* < 0.001.

Next, we measured IFN-γ levels, given previous evidence linking secretion of this proinflammatory cytokine with Gal-9 [14]. We did not observe differences in IFN-γ plasma levels across the follow-up times in HIV+ patients (Figure 2A). However, dispersion of the data was high, suggesting that some patients have higher levels than others. Considering this observation, we divided patients into two groups: those without and those with a reported concomitant infection (2 with syphilis, 16 with molluscum) for further analysis. We observed that HIV+ patients with concomitant infections had a higher IFN-γ level than those without concomitant infections (Figure S1A). Statistical analysis performed only among HIV+ patients without known coinfections showed IFN-γ level decreased at week 8 [16.28, 7.333–43.34, *p* = 0.0247], 12 [25.27, 11.79–41.91, *p* = 0.0271] and 24 [13.46, 6.936–28.5, *p* = 0.0396] compared to week 0 [87.83, 65.16–158.4] (Figure S1B), suggesting that IFN-γ levels decreased after ART initiation only among patients without coinfections. In HIV+IRIS patients, we observed a decrease in IFN-γ levels across the follow-up. IFN-γ levels were lower at each evaluated point, including at the time of the IRIS event [16.6, 8.856–42.21, *p* = 0.0008], than at week 0 [77.13, 56.09–139.8, *p* = 0.0008] (Figure 2B). Of note, two patients showed IFN-γ values up 320-fold higher than the median across the complete follow-up; one of them had IRIS associated with HZV, and the other with HSV (Figure S1C). These patients were excluded in Figure 2A because a statistical analysis (ROUT method, Q = 0.1%) classified them as outliers. 

### 3.4. sTNF-α Was Increased at IRIS Onset

Considering that the median time of IRIS development was 7.5 weeks and results between 12 and 24 weeks were similar for each molecule evaluated so far, we restricted follow-up to 0, 4, 8, and 12 weeks to focus on a time frame closer to the moment of IRIS development. 

Considering previous observations suggesting that proinflammatory cytokines including IL-6 and the soluble form of TNF-α (sTNF-α) were increased during mycobacterial IRIS, and that both molecules have been proposed as possible markers for the prognosis and diagnosis of IRIS [1,15,16], we assessed these molecules in the present study.

**Figure 3:**
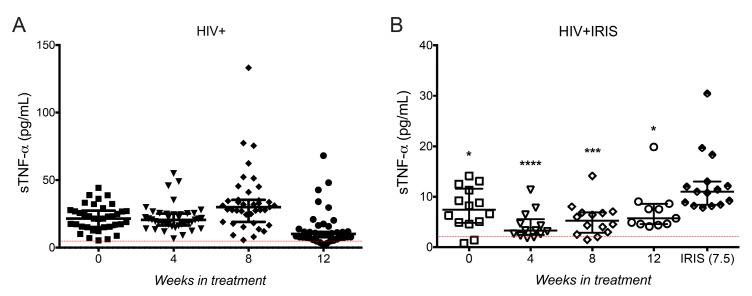
Proinflammatory cytokine TNF-α increased at IRIS events. sTNF-α circulating levels in HIV+ (**A**) at 0- (■), 4- (▼), 8- (♦), 12- (●) weeks after ART, and HIV+IRIS patients (**B**) at 0- (☐), 4- (▽), 8- (◇), 12- (○) weeks after ART, and IRIS episodes (⬘). The red line represents the mean value of healthy donors. Post hoc Dunnett’s test compares the control (0 W) for the HIV+ group or IRIS with the HIV+IRIS group. * *p* < 0.05, *** *p* < 0.001, **** *p* < 0.0001.

sTNF-α levels did not show differences between follow-up times in HIV+ patients (Figure 3A). When we separated patients with and without concomitant infections, only one patient with concomitant infections (condyloma) had a higher sTNF-α level than the rest (Figure S2A). In HIV+ patients without coinfections, sTNF-α increased at week 8 [27.73, 16.88–34.89, *p* = 0.0226] and decreased at week 12 [7.812, 5.766–11.05, *p* = 0.0008] compared with week 0 [23.01, 14.44–26.82] (Figure S2B). 

Interestingly, sTNF-α levels were significantly increased at IRIS episodes [10.89, 8.36–12.34] compared with week 0 [7.395, 4.801–11.59, *p* = 0.0330], 4 [3.286, 2.418–5.528, *p* = 0.0001], 8 [5.238, 2.851–6.87, *p* = 0.0004], and 12 [5.707, 4.56–8.607, *p* = 0.0313] (Figure 3B). Although sTNF-α level at IRIS episodes was higher compared to other follow-up times, it is worth noting that HIV+IRIS patients, as group, showed lower levels than the group of HIV+ patients (Figure 3A,B). Of note, the two patients with higher IFN-γ levels (associated with HZV and HSV infections) also had sTNF-α levels up to 130-fold higher than the median (Figure S2C). Finally, we did not find significant changes in IL-6 plasma levels (Figure S3). In summary, our data suggest that HIV+IRIS patients display lower sTNF-α levels than HIV+ patients; however, sTNF-α levels significantly increase at IRIS onset.

### 3.5. TNF-α Receptors and E-cadherin Were Not Affected at IRIS Events

Both sTIM-3 and sTNF-α are delivered from the cell surface into their soluble forms by the proteolytic activity of members of the metalloproteinase domain-containing protein (ADAM) family. Specifically, TIM-3 is a substrate of ADAM10 and ADAM17, whereas TNF-α is a substrate of ADAM17 [17,18]. In order to assess if the high levels of sTIM-3 and sTNF-α observed in HIV+IRIS patients, specifically at IRIS events, were merely a consequence of a high activation of ADAM proteases and not a specific increase of sTIM-3 and sTNF-α production, we measured several substrates of ADAM10 and ADAM17 proteases in the HIV+IRIS group. TNF receptors (TNFR1 and TNFR2) and E-cadherin are released to their soluble forms by ADAM proteases: sTNFR1 and sTNFR2 by ADAM17, and sE-cadherin by ADAM10 [19,20]. 

**Figure 4:**
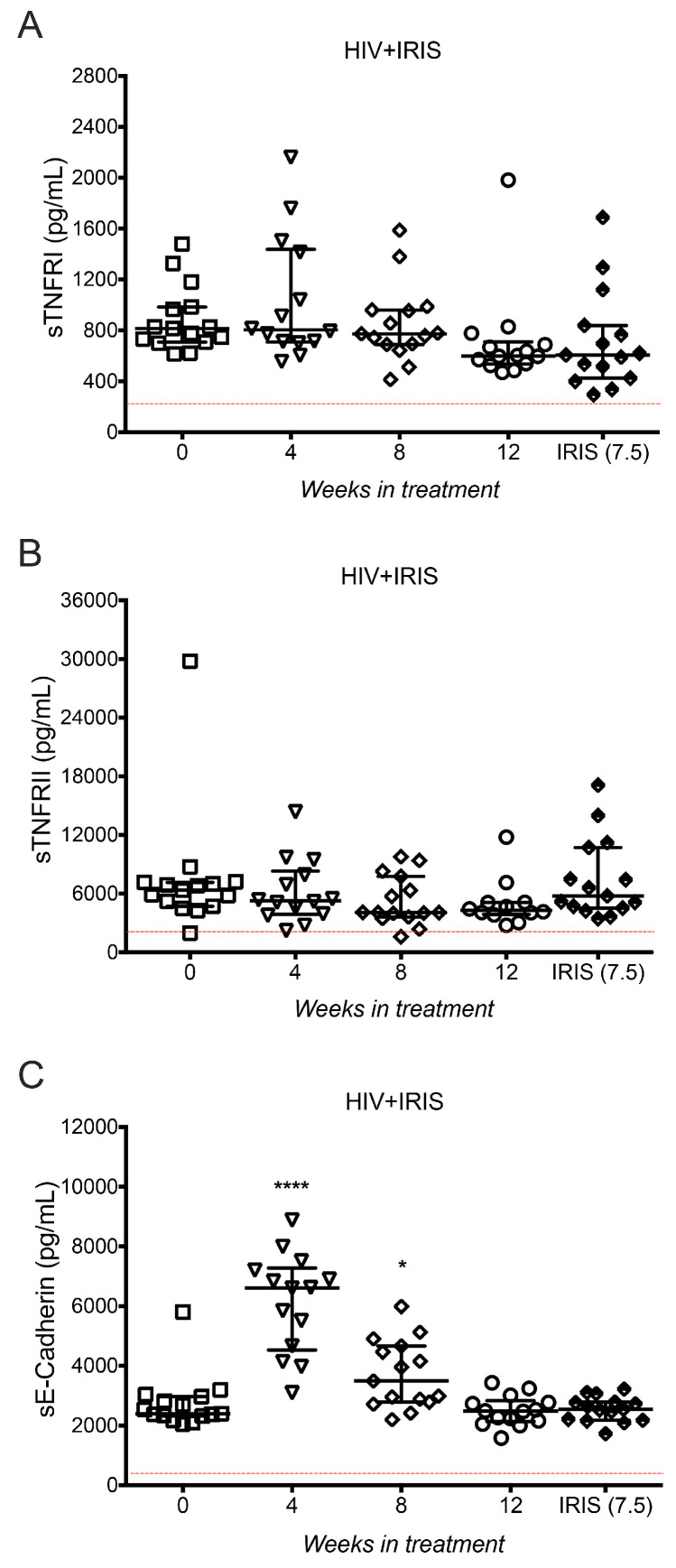
sTNFR1, sTNFR2, and sE-cadherin circulating levels did not increase at IRIS events. sTNFR1 (**A**), sTNFR2 (**B**), and sE-cadherin (**C**) in HIV+IRIS patients at 0- (☐), 4- (▽), 8- (◇), 12- (○) weeks after ART, and IRIS episodes (⬘). The red line represents the mean value of healthy donors. Post hoc Dunnett’s test compares IRIS episodes between the HIV+IRIS group. * *p* < 0.05, **** *p* < 0.0001.

sTNFR1 and sTNFR2 levels did not show a modification across the follow-up compared to the time of IRIS (Figure 4A,B). sE-cadherin level was similar at the time of IRIS and at week 0 [2394, 2321–2967] but increased at week 4 [6612, 4532–7281, *p* = 0.0001], and week 8 [3496, 2789–4665, *p* = 0.0101] compared to the time of IRIS (Figure 4C).

These results suggest that high levels of sTIM-3 and sTNF-α are characteristic of IRIS onset and do not seem to result from overactivation of ADAM proteases, given that other ADAM substrates, such as sTNFR1, sTNFR2, and sE-cadherin, did not increase at IRIS episodes. 

### 3.6. ADAM10 and ADAM17 Did Not Increase at the Onset of IRIS

**Figure 5:**
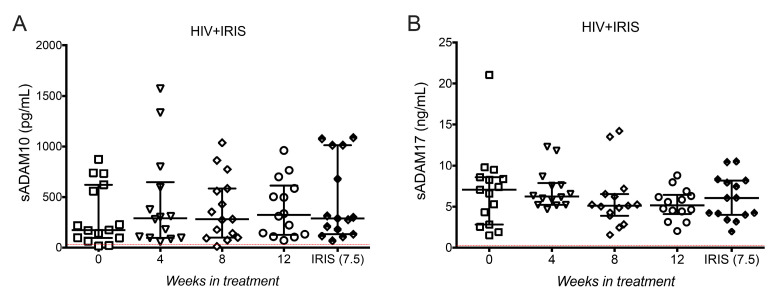
Metalloproteases sADAM10 and sADAM17 did not increase at IRIS events. sADAM10 (**A**) and sADAM17 (**B**) in HIV+IRIS patients at 0- (☐), 4- (▽), 8- (◇), 12- (○) weeks after ART, and IRIS episodes (⬘). The red line represents the mean value of healthy donors. Post hoc Dunnett’s test compares IRIS episodes between the HIV+IRIS group.

As sTNFR1, sTNFR2, and sE-cadherin levels do not exhibit any excessive shedding mediated by ADAM10 and ADAM17 at IRIS episodes; another possible explanation for the high level of sTIM-3 and sTNF-α observed was the overproduction of the proteases themselves. When quantifying ADAM10 and ADAM17 soluble forms in plasma (sADAM10 and sADAM17, respectively) we did not observe variations across the follow-up, including at the time of IRIS (Figure 5A,B).

Together, these data suggest that high levels of sTIM-3 and sTNF-α are elevated in IRIS through mechanisms that are independent of ADAM protease activity.

## 4. Discussion

This study aimed to identify a marker profile at IRIS onset using an accessible biological specimen such as plasma. We measured plasma levels of selected molecules, including cytokines, receptors, and ectoenzymes (ADAM family) to assess changes that could be useful in predicting or diagnosing IRIS. Although IRIS is a life-threatening complication that occurs in some patients starting ART, mostly in the context of late initiation, its diagnosis and prediction continues to be a challenge for a better management of patients. No specific biomarkers have been described that could help in the better understanding and diagnosing IRIS; thus, several studies have been conducted to find immunological or biochemical biomarkers to cover this great need in the field [1,21]. 

Currently, the best-identified risk factors associated with the development of IRIS include a low baseline CD4+ T cell count (<50 cells/μL) and high viral load [21]. The finding of specific IRIS markers has been difficult because IRIS is a diagnosis of exclusion due to the complexity of clinical manifestations in severely immunocompromised patients. Moreover, clinical manifestations of pathogen-specific immune responses in IRIS can vary extensively depending on the type of pathogen [1].

In this work, we evaluated plasma levels of immunological markers in HIV+ infected patients who developed and who did not develop IRIS after late ART initiation. 

Our data showed that sTIM-3 increased at IRIS events, while its ligand, sGal-9, had the opposite behaviour. Since the IRIS events occurred approximately 8 weeks after ART initiation, our data suggest that sTIM-3 increases at the IRIS episodes. This value was different even to adjacent follow-up times (weeks 4 and 8) post-ART initiation. T cell exhaustion is an important mechanism involved in T cell dysfunction in chronic HIV infection; TIM-3 and PD-1 are markers of exhaustion involved in CD4+ T cell loss in untreated chronic HIV infection and CD4 restoration in treated infection [22,23]. An increase in sTIM-3 at IRIS events could be associated with a rapid CD4+ T cell count restoration after ART initiation, suggesting a possible explanation for the presence of the exhausted CD4+ and CD8 T cells at IRIS occurrence. Additional studies of TIM-3 expression are necessary to understand its participation in the immunological response at IRIS events. Even though there are studies suggesting that cell activation is a mechanism occurring in IRIS [2], during IRIS associated with mycobacterial infections, memory CD8+T cells have increased expression of killer cell lectin-like receptor subfamily G member 1 (KLRG1) and programmed death 1 (PD-1), whereas PD-1+CD4+ overexpressed LAG-3 and CTLA-4 T [24]. Moreover, it has been reported that a high frequency of CD8+ T cells is a specific risk factor for mycobacterial IRIS [25]. These data suggest that CD4+ and CD8+ T cells are differentially regulated at IRIS onset. Thus, analysis of TIM-3 expression on T cells of HIV+IRIS patients could be relevant to know more about T cell function and T cell exhaustion in the context of IRIS. Results obtained in this study open new opportunities to investigate if sTNF-a and sTIM-3 play a role in inducing an exhausted T cell profile or possibly affect functions of other cell populations, including both myeloid and lymphoid subpopulations. 

Gal-9, the ligand of TIM-3 plays an essential role in regulating innate and adaptive immune responses in HIV pathogenesis; levels of sGal-9 are increased after HIV infection in association with HIV transcription, despite viral suppression by ART [12]. We found sGal-9 levels in all HIV+ patients were initially increased, decreasing after ART initiation, without reaching similar levels to healthy donors. Even though we did not investigate why sTIM-3 increased at IRIS, while sGal-9 did not, these results suggest that sTim-3 levels can be regulated by an enzymatic mechanism rather than gene expression. However, this is a relevant question that warrants further study, both at the molecular and protein expression level. Although sGal-9 concentration did not increase at the IRIS events, it was associated with IFN-γ. Like Gal-9, IFN-γ plasma levels decreased at IRIS, suggesting that the exacerbated inflammatory response was not due to secretion of IFN-γ. Notably, HIV+ patients with high plasma levels of IFN-γ had clinical events not considered manifestations of IRIS at the moment of assessment. Among 46 HIV+ patients, 25 presented concomitant infections, mainly syphilis and *Molluscum contagiosum*, and an increase of IFN-γ could well be associated with those manifestations (Figure S1A), as suggested by the observation that when these patients were excluded from statistical analysis, IFN-γ levels decreased in response to ART (Figure S1B). Moreover, HIV+IRIS patients with high IFN-γ and sTNF-α levels presented infections with herpes viruses that required hospitalisation and still had detectable HIV viral load at 12 weeks (Figures S1C and S2C). 

IL-6 has been reported as a potential biomarker of IRIS associated with MAC and cryptococcal meningitis (CM) [15,26]. There are multiple proinflammatory cytokine responses at IRIS events; however, only an increase of CRP and IL-6 levels after ART initiation have been associated with an increased risk of IRIS [1,26]. Although CM-IRIS patients were not included in our study, some patients included developed IRIS associated with MAC and TB. Interestingly, IL-6 plasma levels were similar across the follow-up, including at the time of IRIS events. Only one patient with MAC-IRIS kept high concentrations of IL-6 up to week 12 of follow-up. In contrast, it is noteworthy that sTNF-α increased significantly at the IRIS onset, except for one of the patients with HZV-associated IRIS, who had high sTNF-α levels during the first 12 weeks but not at the IRIS episode (Figure S1C). IL-6 was not increased at IRIS, probably because there were no CM-IRIS patients, and only two patients with MAC-IRIS were included in this analysis.

One of the most relevant findings in our cohort was the increase of sTNF-α at IRIS onset. However, additional studies are needed to confirm whether this increase is characteristic of IRIS itself or is a pathogen-associated effect, since the patients that showed a high level of IFN-γ (HZV and HSV-associated IRIS) also had high levels of sTNF-α across the complete follow-up. These patients had a background of *Pneumocystis jiroveci*-MAC and *Pneumocystis jiroveci*-histoplasmosis coinfections. Importantly, the increase of sTNF-α in these two patients did not occur at the time of the IRIS events. Furthermore, the complete group of HIV+ patients had higher sTNF-α levels than the HIV+IRIS group. 

The observation of increased sTIM-3 and sTNF-α levels at IRIS onset led us to propose two hypotheses: (1) other related molecules that are released to their soluble form by the action of ADAM10 or ADAM17 could be increased in the plasma of HIV+IRIS patients, and (2) high sTIM-3 and sTNF-α levels could be directly correlated with an increase of ADAM10 and ADAM17 plasma levels. Membrane TNF-α is cleaved by the metalloprotease TACE (ADAM17), producing soluble TNF-α [18], the unique form of TNF-α found in blood and plasma. Furthermore, TNF receptors play an essential role in the TNF-α pathway, and both soluble and membrane TNF-α bind to the two transmembrane receptor molecules, TNFR1 and TNFR2, which are also substrates for ADAM17 [19]. We found that both sTNFR1 and sTNFR2 increased across the complete follow-up, but there was no significant difference at the time of IRIS onset. The patient who developed MAC-IRIS showed higher levels of both receptors, TNFR1 during the complete follow-up and TNFR2 at weeks 0 and 4. Proteolytic cleavage of E-cadherin mediated by ADAM10 regulates epithelial cell–cell adhesion, migration, and β-catenin translocation [20]. Moreover, a significant redistribution of E-cadherin within the intestinal mucosa during HIV-1-associated disruption of epithelial integrity and an increase of sE-cadherin in the plasma can lead to a direct and substantial abrogation of antiviral functions of HIV-1-specific CD8+ T cells [27]. We found that sE-cadherin increased at weeks 4 and 8, although at week 12 and at the time of the IRIS event, levels were similar to baseline. 

TIM-3 can be cleaved by the action of both ADAM10 and ADAM17 depending on the activation signal [17]. Currently, sTIM-3 is poorly characterised, and its participation in immune responses is not well understood. Nevertheless, it has been shown that sTIM-3 levels increase significantly during early and chronic untreated HIV infection. Interestingly, sTIM-3 shedding from CD8+ T cells by ADAM10 increases its levels in plasma during untreated HIV infection and correlates with HIV disease progression [28]. Finally, we measured ADAM10 and ADAM17 plasma levels and found that they did not change across the follow-up, and that they were not affected at the time of IRIS events.

This study has some limitations: (1) it only included male patients, excluding a minority of female patients from the source cohort, and (2) the sample size was small (15 HIV+IRIS patients). Nevertheless, both study populations have been thoroughly characterised and followed up for a long time, allowing us to ensure that our groups are comparable regarding baseline characteristics. Therefore, this cohort is suitable for our main objective: looking for specific biomarkers of IRIS. Indeed, sTIM-3, sGal-9, and IFN-γ levels were as expected in the HIV+ patients, and patients showed a favourable response to ART with regard to both viral load and CD4 T cells counts recovery at week 24 [9].

## 5. Conclusions

In conclusion, we have shown that sTIM-3 and sTNF-α were increased in patients who developed IRIS and could be considered characteristic IRIS biomarkers. The mechanisms by which these molecules are involved in IRIS warrant further investigation. Our data suggest that sTIM-3 and sTNF-α could be used as IRIS markers as an additional aid in clinical diagnosis. The determination of these two markers in plasma could add value to clinical management and requires minimal intervention, as only an easily accessible peripheral blood specimen is required. 

## Figures and Tables

**Table 1:** Characteristics of non-IRIS and IRIS patients.

Parameters	All, *n* = 61	HIV+, *n* = 46 (75%)	HIV+IRIS, *n* = 15, (25%)
Age (median) IQR	34 (29–39)	32 (29–39)	36 (29–40)
**CD4 count cells/ml, median (IQR)**			
Week 0	55 (31–105)	54 (31–124)	58 (25–101)
Week 4 post-ART	151 (79–215)	132 (75–214)	165 (146–217)
Week 8 post-ART	159 (117–252)	158 (122–256)	160 (113–215)
Week 12 post-ART	164 (102–231)	166 (104–247)	160 (100–230)
Week 24 post-ART	199 (152–274)	204 (148–274)	179 (161–274)
**CD4/CD8 ratio, median (IQR)**			
Baseline	0.07 (0.04–0.13)	0.07 (0.04–0.13)	0.08 (0.03–0.13)
Week 4 post-ART	0.14 (0.09–0.25)	0.14 (0.10–0.25)	0.13 (0.08–0.28)
Week 8 post-ART	0.17 (0.1–0.25)	0.17 (0.11–0.24)	0.14 (0.09–0.25)
Week 12 post-ART	0.17 (0.11–0.26)	0.18 (0.12–0.27)	0.15 (0.11–0.22)
Week 24 post-ART	0.21 (0.14–0.29)	0.21 (0.14–0.28)	0.20 (0.12–0.31)
**HIV Viral load, Log_10_ copies/ml, median (IQR)**			
Baseline	5.57 (5.1–5.8)	5.5 (5.1–5.8)	5.8 (5.2–6.0)
Week 4 post-ART	2.8 (2.6–3.1)	2.8 (2.6–3.1)	2.8 (2.5–3.1)
Week 8 post-ART	2.2 (1.6–2.6)	2.2 (1.8–2.6)	2.0 (1.6–2.6)
Week 12 post-ART	1.8 (1.6–2.6)	1.9 (1.6–2.6)	1.7 (1.6–2.6)
Week 24 post-ART	1.6 (1.6–1.6)	1.6 (1.6–1.6)	1.6 (1.6–1.6)

Mann–Whitney U test was performed between HIV+ vs. HIV+IRIS groups.

**Table 2:** Characteristics of subjects developing IRIS.

Parameters	*n* = 15
Time to IRIS onset, weeks, median (IQR)	7.5 (4.1–19.0)
Manifestations related with IRIS	
Mycobacteria, *n* (%)	4 (27%)
*Mycobacterium tuberculosis*	2
*Mycobacterium avium complex*	2
Herpesviruses, *n* (%)	7 (47%)
*Herpes Zoster*	4
*Herpes Simplex Virus*	2
*Cytomegalovirus*	1
Other dermatological manifestations, *n* (%)	3 (20%)
*Molluscum contagiosum*	1
Eosinophilic folliculitis	2
Lymphadenopathy not specified, *n*(%)	1 (6%)

Data are shown as percentages.

## Data Availability

All data relevant to the study are included in the article or uploaded as supplementary information. Authors confirm that the raw data to support the conclusions of this study are included in the manuscript. Corresponding author will provide more information, upon rational request, to any qualified researcher.

## Supplementary Materials

The following are available online at https://www.mdpi.com/article/10.3390/life11060527/s1, Figure S1: Circulating levels of IFN-γ in HIV+ and HIV+IRIS patients, Figure S2: Circulating levels of TNF-α in HIV+ and HIV+IRIS patients, Figure S3: Proinflammatory cytokine IL-6 was not affected at IRIS events, Table S1: Plasma levels of all studied molecules in healthy donors.
