# Supplementary material for: High Levels of TNF-α and TIM-3 as a Biomarker of Immune Reconstitution Inflammatory Syndrome in People with HIV Infection
Source: Life (Basel). 2021 Jun 5;11(6):527. doi: 10.3390/life11060527 (PMC8227006; doi:10.3390/life11060527)
Supplement: Supplementary file 1 [file life-11-00527-s001.zip › life-1206151-supplementary.pdf]

life

MDPI

Article

High Levels of TNF-α and TIM-3 as a Biomarker of Immune Reconstitution Inflammatory Syndrome in People with HIV Infection

Lucero A. Ramon-Luing[1], Ranferi Ocaña-Guzmán[1], Norma A. Téllez-Navarrete[1], Mario Preciado-García[1], Dámaris P. Romero-Rodríguez[2], Enrique Espinosa[1], Gustavo Reyes-Terán[3], and Leslie Chavez-Galan[1]*

[1] Laboratory of Integrative Immunology, Instituto Nacional de Enfermedades Respiratorias "Ismael Cosío Villegas", Mexico City, Mexico; ramonluing@yahoo.com.mx (LARL), ranferi.og@gmail.com (ROG), norma.tellez@gmail.com (NATN), mario77.preciado@gmail.com (MPG), hector.enrique.espinosa@gmail.com (EE).

[2] Flow Cytometry Core Facility, Instituto Nacional de Enfermedades Respiratorias "Ismael Cosío Villegas", Mexico City, Mexico; damaquim@gmail.com (DPRR).

[3] Center for Infectious Diseases Research (CIENI), Instituto Nacional de Enfermedades Respiratorias "Ismael Cosío Villegas", Mexico City, Mexico; gustavo.reyesteran@gmail.com (GRT).

* Correspondence: lchavezgalan@gmail.com; lchavez_galan@iner.gob.mx (L. C.-G.). +525554871700 X. 5270

Supplementary Material

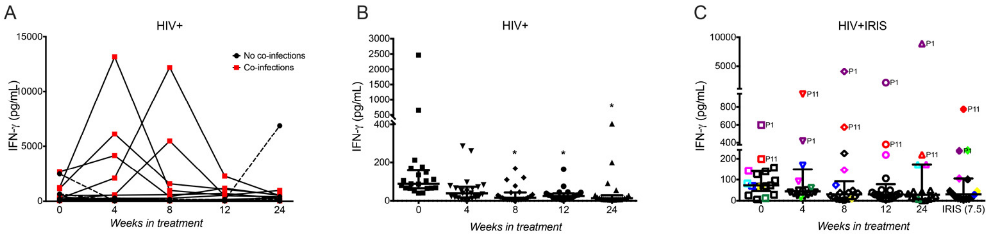

Figure S1. Circulating IFN-γ levels in (**A**) all HIV+ patients, red squares represent patients with concomitant coinfections (syphilis and molluscum), and black circles represent patients with no coinfections. (**B**) Analysis excluding HIV+ patients with concomitant infections. (**C**) IFN-γ in HIV+IRIS patients at 0-, 4-, 8-, and 12-weeks after ART and IRIS episodes. Post hoc Dunnett's test compares successive follow-up times with 0 weeks.

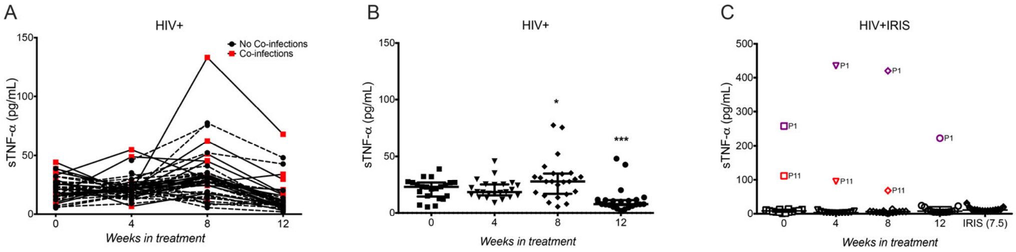

Figure S2. Circulating sTNF-α levels in (**A**) all HIV+ patients, red squares represent patients with concomitant coinfections (syphilis and molluscum), and black circles represent patients with no coinfections. (**B**) Analysis excluding HIV+ patients with concomitant infections. (**C**) sTNF-α in HIV+IRIS patients at 0-, 4-, 8-, and 12-weeks after ART and IRIS episodes. Post hoc Dunnett's test compares successive follow-up times with 0 weeks.

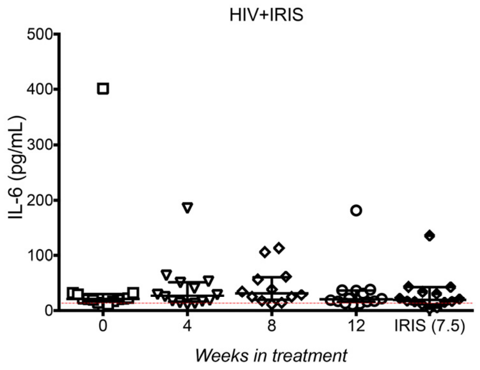

Figure S3. Proinflammatory cytokine IL-6 was not affected at IRIS events. IL-6 plasma levels in HIV+IRIS patients at 0-, 4-, 8-, and 12-weeks after ART and IRIS episodes. The red line represents the mean value from 10 healthy donors. Post hoc Dunnett's test was used to compare with IRIS episodes.

Table S1. Mean of plasma levels of all studied molecules evaluated in healthy donors.

Molecule	pg/mL (mean)	n (healthy donors)
TIM-3	2514.0	6 [1]
Gal-9	1912.0	6 [1]
IFN-γ	1.8.0	6 [1]
TNF-α	4.1.0	6 [1]
TNFR1	253.1	16 [2]
TNFR2	549.5	23 [2]
ADAM10	5.0	6 [2]
ADAM17	120.8	23 [2]
E-Cadherin	336.7	10 [2]
IL-6	9.68	10 [2]

Value previously reported by our group [1] [11]. Unpublished data obtained in our laboratory [2].
